# Years of Potential Life Lost Because of Cardiovascular Disease in Asian‐American Subgroups, 2003–2012

**DOI:** 10.1161/JAHA.118.010744

**Published:** 2019-03-20

**Authors:** Divya G. Iyer, Nilay S. Shah, Katherine G. Hastings, Jiaqi Hu, Fatima Rodriguez, Derek B. Boothroyd, Aruna V. Krishnan, Titilola Falasinnu, Latha Palaniappan

**Affiliations:** ^1^ University of Connecticut School of Medicine Farmington CT; ^2^ Division of Primary Care and Population Health Stanford University School of Medicine Stanford CA; ^3^ Division of Cardiovascular Medicine Stanford University Stanford CA; ^4^ Quantitative Sciences Unit Stanford University School of Medicine Stanford CA; ^5^ Division of Epidemiology Department of Health Research & Policy Stanford University School of Medicine Stanford CA

**Keywords:** disparities, ethnicity, heart disease, life expectancy, premature mortality, stroke, Cardiovascular Disease, Race and Ethnicity, Mortality/Survival, Epidemiology, Women

## Abstract

**Background:**

Asian‐American subgroups (Asian‐Indian, Chinese, Filipino, Korean, Japanese, and Vietnamese) display varied cardiovascular disease mortality patterns, especially at younger ages. This study aims to examine the years of potential life lost because of ischemic heart disease and cerebrovascular disease among the 6 largest Asian‐American subgroups compared with non‐Hispanic whites.

**Methods and Results:**

We used National Center for Health Statistics Multiple Causes of Death mortality files from 2003 to 2012 to calculate race‐specific life expectancy, mean years of potential life lost, and years of potential life lost per 100 000 population for each Asian subgroup and non‐Hispanic whites. Asian‐American subgroups display heterogeneity in cardiovascular disease burden. Asian‐Indians had a high burden of ischemic heart disease; Asian‐Indian men lost 724 years per 100 000 population in 2012 and a mean of 17 years to ischemic heart disease. Respectively, Vietnamese and Filipino men and women lost a mean of 17 and 16 years of life to cerebrovascular disease; Filipino men lost 352 years per 100 000 population in 2012. All Asian subgroups for both sexes had higher years of life lost to cerebrovascular disease compared with non‐Hispanic whites.

**Conclusions:**

Cardiovascular disease burden varies among Asian subgroups, and contributes to greater premature mortality in certain subgroups. Asian‐Indian and Filipino populations have the highest years of life lost because of ischemic heart disease and Filipino and Vietnamese have the highest years of life lost because of cerebrovascular disease. Analysis of risk factors and development of subgroup‐specific interventions are required to address these health disparities.

**Clinical Perspective**

**What Is New?**

- The burden of premature mortality from cardiovascular disease varies among the 6 largest Asian‐American subgroups.
- Asian‐Indian, Filipino, and Vietnamese populations experience the greatest years of life lost from heart attacks and stroke.

**What Are the Clinical Implications?**

- This research highlights the importance of disaggregating Asian populations to more comprehensively understand the burden of premature cardiovascular mortality.

## Introduction

Asian‐Americans are the fastest growing racial/ethnic group in the United States, having experienced a 46% increase in population from 2000 to 2010, and with an expected population of 34 million by the year 2050.^1^ National mortality surveys have only recently in 2003 separated the 6 largest Asian‐American subgroups (Asian‐Indians, Chinese, Filipinos, Japanese, Korean, and Vietnamese).^2^ This disaggregation has allowed study of individual Asian subgroups, revealing substantial heterogeneity in disease patterns.^3^, ^4^, ^5^, ^6^ Notably, the leading cause of death varies among Asian‐American subgroups.^6^ Heart disease is the leading cause of death in Asian‐Indian, Filipino, and Japanese men.^6^ Asian‐Indian men and women, and Filipino men have a greater proportionate mortality because of ischemic heart disease (IHD) compared with other racial groups and subgroups.^1^ The third leading cause of death in men, among all Asian subgroups, is stroke.^6^

Furthermore, studies of cardiovascular risk factors and age of onset of first acute myocardial infarction have suggested that certain Asian‐American subpopulations have cardiovascular disease (CVD) at a younger age.^7^, ^8^, ^9^, ^10^ This finding raises concern that CVD may contribute to premature mortality in Asian‐American populations. While previous studies^1^ showed greater proportionate mortality because of CVD in Asian‐Indians, this methodology (proportion of deaths because of CVD over total deaths in Asian Indians) does not provide differential weight to premature deaths that occur among younger people.

Preventing premature mortality is an important public health goal. Premature mortality can be quantified by measuring years of potential life lost (YPLL).^11^, ^12^ YPLL compares age at death with average life expectancy to estimate the average time an individual would have lived had he/she not died prematurely from a specific disease.^13^ YPLL is a superior measure of premature mortality when compared with age‐adjusted mortality rate because it can be used to demonstrate the social and economic burden of disease in a population.^13^ YPLL has been proposed as a useful outcome measure in cardiovascular medicine, specifically, because it avoids some important limitations of more commonly used measures such as overall population death rate and case‐fatality rate.^14^

An analysis of YPLL in all Americans revealed acute myocardial infarction as one of the top 10 causes of YPLL, and acute cerebrovascular disease as one of the top 5 causes of YPLL.^15^ However, an analysis of YPLL because of CVD in the diverse and rapidly growing Asian‐American population specifically has not yet been conducted. In this study, we have evaluated disease burden in Asian‐American subgroups by calculating the YPLL because of IHD and cerebrovascular disease. In this analysis, the age at death because of IHD and cerebrovascular disease is compared with Asian‐specific life expectancy, calculated by the US Census Bureau projections.^16^

The aim of our study is to use YPLL to better characterize the burden of CVD in each Asian‐American subgroup. To our knowledge, this is the first analysis of YPLL from CVD in Asian‐Americans using national‐level data.

## Methods

The data that support the findings of this study are available from the first author upon request.

### Study Population

Asian‐American individuals were identified in US mortality records from the National Center for Health Statistics Multiple Causes of Death mortality files from 2003 to 2012. The Stanford University institutional review board provided a waiver for use of these publicly available mortality and census data. We used death records from states that adopted the 2003 revision of the US Standard Certificate of Death, which disaggregated Asian‐American subgroups by providing a check box for the 6 largest subgroups. The following 36 of 51 states that adopted this revision were included in this study: Arizona, Arkansas, California, Connecticut, Delaware, Florida, Georgia, Idaho, Illinois, Indiana, Iowa, Kansas, Kentucky, Maine, Michigan, Minnesota, Missouri, Montana, Nebraska, Nevada, New Hampshire, New Jersey, New Mexico, New York, North Dakota, Ohio, Oklahoma, Oregon, Rhode Island, South Carolina, South Dakota, Texas, Utah, Vermont, Washington, and Wyoming. Death certificate data from National Center for Health Statistics provided cause of death, race/ethnicity of the decedent, and participant's age at death. *International Classification of Diseases Tenth Revision* (*ICD‐10*) codes I20‐I25 identified death from IHD, and codes I60‐I69 identified death from cerebrovascular disease. An aggregate Asian category comprised data from the 6 main Asian‐American groups used in this analysis.

### Statistical Analysis

Descriptive statistics were calculated in each Asian‐American subgroup, including total decedents, sex distribution, total deaths from IHD and cerebrovascular disease, percent of deaths because of IHD and cerebrovascular disease, mean age at death for both diseases with standard deviation, percent of each population born in the United States, percent born in a foreign country, and distribution of highest education level attained.

Race‐specific life expectancy was calculated using the most recent available Census data on Asian life expectancy during the study period 2003 to 2012.^16^ Linear interpolation was used to estimate the life expectancy of Non‐Hispanic Asian and Non‐Hispanic white (NHW) populations for 2007, the midpoint year of the study period. Non‐Hispanic Asian life expectancy for the year 2007 was calculated to be 81 for men, and 87 for women. NHW life expectancy for the year 2007 was calculated to be 76 for men, and 81 for women. Life expectancy was also calculated for the final year in the study period, 2012, and was found to be 82 for Non‐Hispanic Asian men and 87 for Non‐Hispanic Asian women, and 76 for NHW men and 82 for NHW women. Linear interpolation is an appropriate method to estimate race‐specific life expectancy from the limited available data on Asian‐American mortality, and has been used in prior study to obtain Asian‐American demographic information.^17^

Mean YPLL was calculated to estimate YPLL because of IHD and cerebrovascular disease for an individual patient, and to compare differences across Asian subgroups. This analysis was completed by subtracting mean age at death from race‐specific life expectancy, and included deaths that occurred before the average life expectancy for that subgroup.

In order to compare differences in the burden of disease between each subgroup population and in comparison to the NHW population, YPLL in years per 100 000 individuals was calculated based on standard epidemiologic methods.^13^ Mean age at death was subtracted from a reference age, and was standardized and age‐adjusted (Data S1). The reference age used in this analysis was race‐specific life expectancy, as calculated above. Each Asian subgroup was compared with the aggregated Asian‐specific life expectancy, and NHW populations were compared with the NHW‐specific life expectancy.

YPLL was standardized and age‐adjusted to allow comparison between each cause of death within each race, sex, and year group. The formula used for standardization and age‐adjustment utilized age categories and took into account percentage of total individuals in each age category. This method is based on standard techniques used for the calculation of YPLL when comparing groups with varying age structures, as is the case among Asian‐American subpopulations and NHW populations.^18^

## Results

From 2003 to 2012, there were a total of 354 256 Asian‐American decedents aged 25 years or older. In this group, 59 936 deaths were because of IHD and 28 489 deaths were because of cerebrovascular disease. Mean age at death varied across each Asian‐American subgroup and with respect to the aggregated Asian population estimate (Table). Mean age at death from IHD was lowest for Asian‐Indians, 71.8 years and highest in Japanese, 81.1 years. Vietnamese decedents had the lowest mean age at death because of cerebrovascular disease, 72.5, followed by Asian‐Indians and Filipinos, while Japanese had the highest mean age at death, 81.4 years.

**Table 1:** Decedent Characteristics, 2003–2012

	Asian Indian	Chinese	Filipino	Japanese	Korean	Vietnamese	Aggregate Asian	NHW
Total decedents	37 779	102 435	86 921	66 235	32 823	28 063	354 256	19 722 445
Sex (% female)	41.0	47.4	49.1	54.7	53.2	43.5	48.7	51.0
Total IHD deaths, %	8431 (22.3)	16 835 (16.4)	15 688 (18.0)	10 347 (15.6)	4931 (15.0)	3704 (13.2)	59 936 (16.9)	3 400 187 (17.2)
Mean age (y) at death because of IHD (SD), 2003–2012	71.8 (13.6)	80.9 (12.4)	76.5 (13.6)	81.1 (11.7)	78.5 (13.3)	74.1 (14.6)	77.9 (13.4)	78.3 (13.4)
Mean age (y) at death because of IHD (SD), 2012	73.5 (13.3)	81.5 (12.8)	76.4 (14.2)	82.7 (11.7)	78.7 (14.1)	75.2 (15.3)	78.4 (13.8)	78.1 (13.7)
Total cerebrovascular disease deaths, %	2249 (6.0)	8340 (8.1)	7633 (8.8)	5319 (8.0)	2409 (7.3)	2539 (9.0)	28 489 (8.0)	1093 163 (5.5)
Mean age (y) at death because of cerebrovascular disease (SD), 2003–2012	72.8 (14.8)	79.3 (13.1)	74.3 (14.8)	81.4 (11.7)	74.5 (15.1)	72.5 (15.1)	76.8 (14.2)	80.8 (12.3)
Mean age (y) at death because of cerebrovascular disease (SD), 2012	73.5 (14.4)	80.5 (13.2)	74.4 (15.2)	81.99 (11.9)	74.7 (14.4)	74.2 (15.3)	77.4 (14.4)	80.8 (12.6)
US born, %	8.0	13.1	11.3	74.9	6.2	4.9	22.4	93.9
Foreign born, %	90.5	86.5	88.2	24.7	88.3	94.3	76.6	5.0
Education—less than high school, %	28.0	36.2	24.4	19.0	29.2	36.0	28.5	23.3
Education—high school only, %	33.8	36.4	41.6	62.2	39.2	45.8	43.3	58.2
Education—college graduate, %	35.5	24.8	32.3	16.3	28.6	15.0	25.7	14.6
Education—unknown, %	2.7	2.6	1.7	2.4	3.0	3.2	2.4	3.9

IHD indicates ischemic heart disease; NHW, non‐Hispanic white.

### Mean YPLL

**Figure 1:**
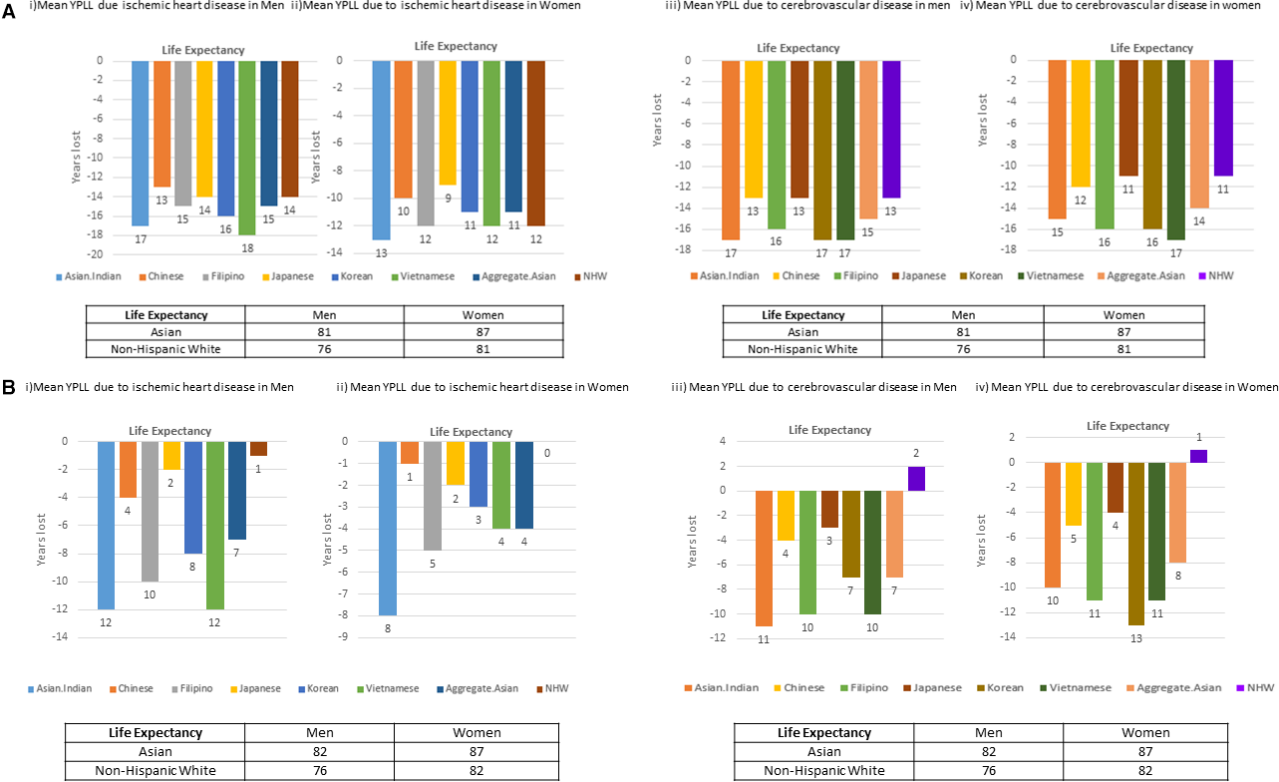
**A**, Mean years of potential life lost (YPLL) because of cardiovascular disease in Asian‐American subgroups, 2003–2012. **B**, Mean years of YPLL because of cardiovascular disease in Asian‐American subgroups, 2012. NHW indicates non‐Hispanic white.

Mean YPLL because of IHD and cerebrovascular disease is depicted in Figure 1A and gives an estimate of the average years lost for a patient who died prematurely in that subgroup during the study period, 2003 to 2012. For ischemic heart disease, Asian‐Indian men and Vietnamese men had the highest mean YPLL, 17 and 18 years, respectively, while Japanese men had a mean YPLL comparable to NHWs, ≈14 years (Figure 1A‐i). Asian‐Indian women on average lost 13 years, followed by Vietnamese and Filipino women who lost an average of 12 years. Similar to men, Japanese and Chinese women had the lowest mean YPLL because of IHD (Figure 1A‐ii). For comparison, mean YPLL was calculated for Asians as an aggregate group to yield 15 years of mean YPLL for men, and 11 years for women. Mean YPLL was also calculated for the final year in the study period, 2012, and is depicted in Figure 1B. Asian‐Indian and Vietnamese men again had the highest mean YPPL, 12 years, while NHW men had a mean YPLL of 1 year (Figure 1B‐i). Asian‐Indian women in the year 2012 had a mean YPLL of 8 years (Figure 1B‐ii).

Calculation of mean YPLL because of cerebrovascular disease during the study period 2003 to 2012 also revealed distinct differences between Asian subgroups. Asian‐Indian, Vietnamese, and Korean men had the highest YPLL because of cerebrovascular disease, 17 years, followed by Filipino men, 16 years (Figure 1A‐iii). Vietnamese women had the highest mean YPLL, 17 years, followed by Korean and Filipino women, who lost an average of 16 years because of cerebrovascular disease (Figure 1A‐iv). Mean YPLL because of cerebrovascular disease in the Asian population as an aggregate was lower in total than when it was calculated specifically for certain subgroups; the mean YPLL was 15 years for men and 14 years for women. Additionally, NHW men and women had much lower mean YPLL because of cerebrovascular disease, 13 and 11 years, respectively. Mean YPLL from the year 2012 was calculated to be 11 for Asian‐Indian men, and 10 for Filipino and Vietnamese men (Figure 1B‐iii). Korean women lost 13 years, while Filipino and Vietnamese women lost 11 years to cerebrovascular disease (Figure 1B‐iv).

### Ischemic Heart Disease

**Figure 2:**
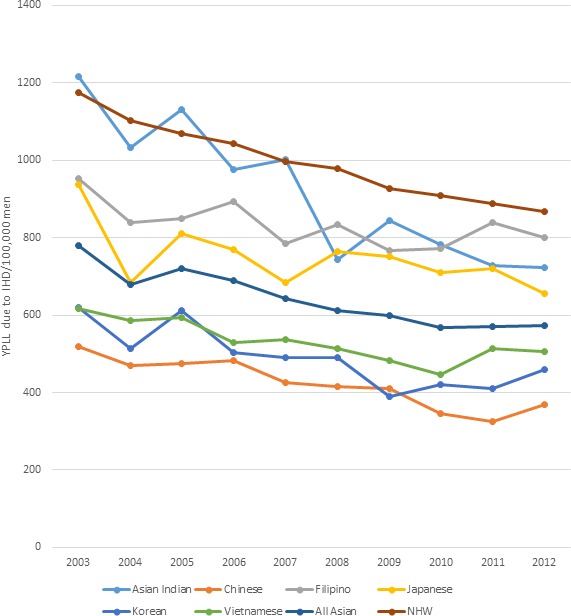
Standardized age‐adjusted years of potential life lost (YPLL) per 100 000 because of ischemic heart disease (IHD) in men (2003–2012).

Using race‐specific life expectancy to calculate YPLL because of IHD, Asian men in aggregate lost 779 years per 100 000 people in 2003, which decreased to 574 years per 100 000 people in 2012. Disaggregation of Asian male subgroups showed a range of YPLL because of IHD across the study period (Figure 2). In 2003, Asian‐Indian men lost 1216 years per 100 000 people, which was higher than all other Asian male subgroups and higher than NHW men. To further characterize the impact of IHD on premature mortality in the Asian‐Indian population, we calculated the proportion of YPLL because of IHD compared with total YPLL from all causes. The proportion of YPLL because of IHD ranged from 25% to 16% over the course of the study period.

By 2012, Filipino‐American men had the highest YPLL compared with the other Asian subgroups, 799 years per 100 000; similar to NHW men in the same year, 807 YPLL per 100 000 population. Furthermore, use of race‐specific life expectancy revealed greater heterogeneity in YPLL across all Asian subgroups. Japanese and Filipino men experienced more YPLL than the aggregate Asian men, while Vietnamese and Korean men experienced fewer YPLL than the aggregate.

**Figure 3:**
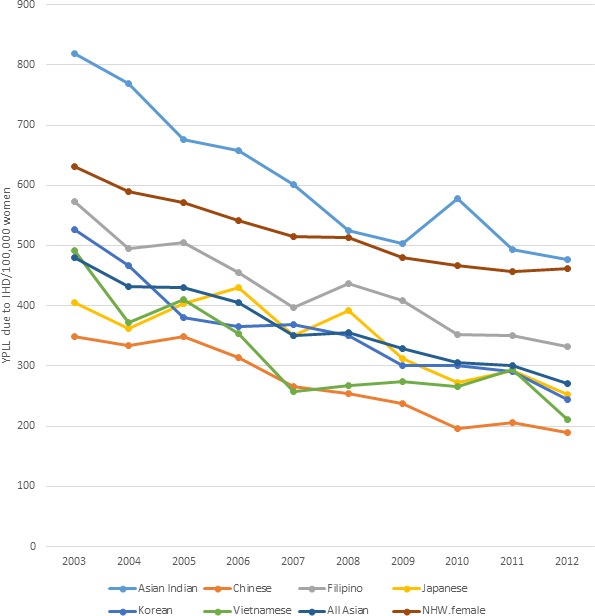
Standardized age‐adjusted years of potential life lost (YPLL) per 100 000 because of ischemic heart disease (IHD) in women (2003–2012). NHW indicates non‐Hispanic white.

In women, trends in YPLL because of IHD were similar to those in men. Figure 3 shows that Asian‐Indian women had the highest YPLL consistently over the study period, ranging from 818 years per 100 000 in 2003, to 477 years per 100 000 in 2012. In contrast, NHW women ranged from 577 years per 100 000 to 426 years per 100 000. Chinese women, like Chinese men, had the lowest YPLL because of IHD, ranging from 348 years per 100 000 to 189 years per 100 000 at the end of the study period.

### Cerebrovascular Disease

**Figure 4:**
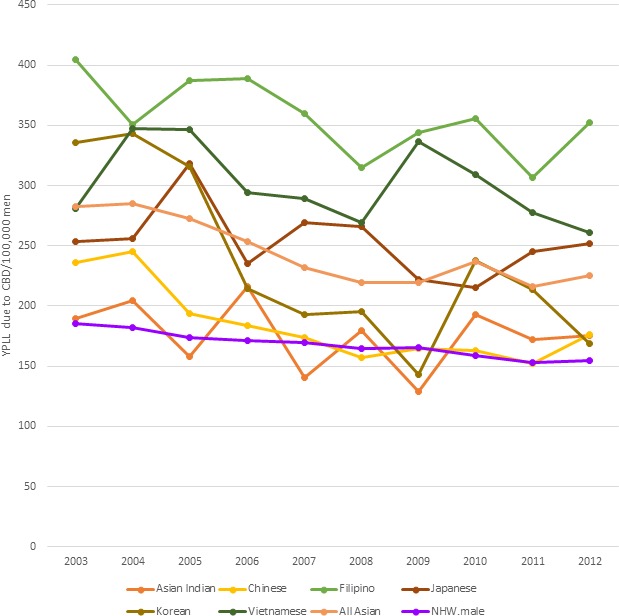
Standardized age‐adjusted years of potential life lost (YPLL) per 100 000 because of cerebrovascular disease (CVD) in men (2003–2012). NHW indicates non‐Hispanic white.

Men in every Asian subgroup lost more years of life to cerebrovascular disease than NHW men. Filipino men had the highest values for YPLL consistently across the study period ranging from 404 years per 100 000 in 2003 to 352 years per 100 000 in 2012, which was higher than NHW males; 170 per 100 000 in 2003 to 143 per 100 000 in 2012 (Figure 4). Vietnamese men had the second highest cerebrovascular disease burden, peaking in the middle of the study period, 2009, when this population lost 337 years per 100 000 individuals, which decreased to 261 years per 100 000 in 2012.

**Figure 5:**
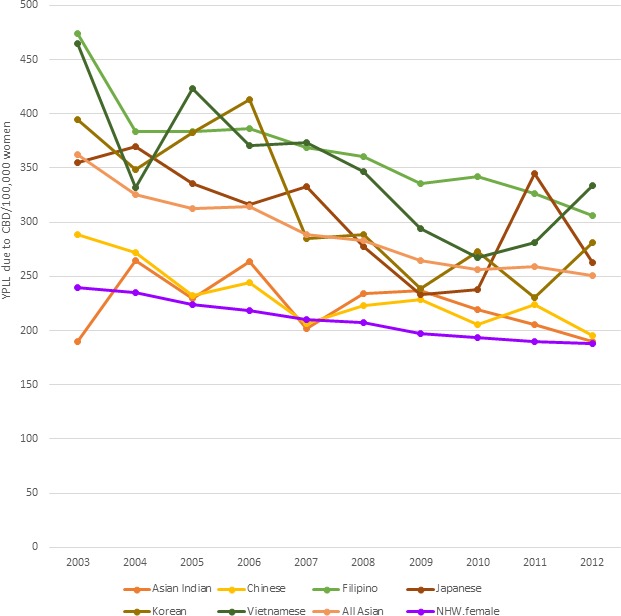
Standardized age‐adjusted years of potential life lost (YPLL) per 100 000 because of cerebrovascular disease (CVD) in women (2003–2012). NHW indicates non‐Hispanic white.

YPLL because of cerebrovascular disease in women was higher for women in each Asian subgroup when compared with NHW women (Figure 5). Filipino and Vietnamese women had the highest YPLL during the study period, similar to the pattern seen in men. Filipino women lost 474 years per 100 000 in 2003, and 306 years per 100 000 in 2012. Chinese and Asian‐Indian women lost fewer years of life than the aggregate Asian estimate, but still more than NHW women during the study period. Chinese women lost 288 years per 100 000 in 2003, and 195 years per 100 000 in 2012, while the aggregate Asian estimate was 362 years per 100 000 in 2003, and 250 years per 100 000 in 2012.

## Discussion

Our analysis of YPLL in Asian‐Americans reveals substantial heterogeneity in the burden of CVD among Asian‐American subpopulations and varied years of life lost at the level of the individual patient.

Asian‐Indians in the study population had a high burden of years lost to IHD and the possibility of losing a decade of life or more to this disease. For this population, almost one fourth of total years lost to any disease were because of IHD, revealing the notable contribution of IHD to overall premature mortality in Asian‐Indians. There are several possible explanations for the greater number of premature IHD deaths among Asian‐Indians. South Asians experience higher death rates from CVD at younger ages and demonstrate higher levels of traditional risk factors at younger ages; a study by Joshi et al found the prevalence of elevated apolipoprotein B_100_/apolipoprotein A‐1 ratio in South Asians to be 61.5% compared with 48.3% in patients from other countries.^7^, ^19^, ^20^, ^21^, ^22^. Type 2 diabetes mellitus is especially important to consider as a contributor to early CVD development and mortality since South Asians tend to develop diabetes mellitus at younger ages.^23^ Additionally, cardiovascular risk in Asian‐Indians may go unnoticed in the clinical setting, and therefore manifest as CVD at a relatively younger age. Support of this notion includes the finding that normal‐weight South Asians have a high prevalence of cardiometabolic abnormalities^24^ and that traditional risk calculation tools, such as the Framingham Risk Score, underestimate risk for Asian Indians.^25^ A recent study also found that South Asian patients have signs of cardiac aging earlier in life, which may contribute to the increased CVD mortality.^26^

Regarding cerebrovascular disease, our study builds upon the prior finding that from 2003 to 2010, every Asian subgroup, except Asian‐Indians, had higher mortality rates and greater proportionate mortality because of hemorrhagic stroke than NHWs.^1^ Our analysis of YPLL found that specifically Filipino and Vietnamese populations lost more years of life than the aggregate Asian population and the NHW population studied. Our findings of high YPLL because of cerebrovascular disease in Filipino populations is consistent with a prior study that has demonstrated increased risk for intracerebral hemorrhage in Filipino patients, which could be explained by increased risk of hypertension in this population, with an odds ratio for hypertension of 1.4 for Filipino men and 1.8 for Filipino women.^27^, ^28^

We present novel measures of the burden of cerebrovascular disease in Vietnamese‐Americans, the study of which is currently very limited. The Vietnamese population in the United States grew 40% from 2000 to 2010 and is currently the fourth largest Asian subgroup, with large communities in certain southern and western states.^29^ Vietnamese communities have a unique migration history, cultural practices, and risk factors warranting more specific attention from the medical community at large. A study found Vietnamese patients had worse self‐reported health status when compared with NHW, possibly because of lower rates of English language proficiency and/or immigration trauma, as many Vietnamese came to the United States as war refugees.^30^ The burden of cerebrovascular disease may be because of increased risk factors such as high rates of smoking among Vietnamese men, with a prevalence of 29.8% in Vietnamese men compared with 19.0% in NHW men, or lower health literacy regarding stroke; only 67% of Vietnamese patients knew that numbness or weakness of the face, arms, or legs was a symptom of stroke.^31^ This combination of risk factors, history, and disease burden highlight a distinct health disparity in the Vietnamese‐American population.

### Study Impact

Our use of race‐specific life expectancy emphasizes the need to understand determinants of health and evaluate health outcomes of Asian‐Americans as a disaggregated group. Asian subgroups have vastly different immigration histories, level of acculturation, risk factors, genetic markers, and cultural practices related to health care.^32^, ^33^, ^34^, ^35^

Our study contributes to the ongoing discussion of whether Asian‐Americans benefit from a mortality advantage when compared with NHW populations. The “model minority” stereotype of Asian‐Americans having higher socioeconomic status and education levels, and lower mortality rates compared with NHWs continues to persist.^36^ Conversely, a study from 2017 found that in certain regions of the United States, some Asian populations (Filipino and Japanese men, Korean and Vietnamese men and women) possessed no mortality advantage in the setting of cerebrovascular disease.^4^ A study in Canada found that IHD in South Asian women was the leading cause of avoidable mortality, and that South Asian women have a disadvantage relative to Canadian‐born women.^37^ Our study shows that a substantial number of Asian patients with CVD, especially Asian‐Indians, Filipinos, and Vietnamese, have a lower age at death and more years of life lost to the disease, meaning they actually experience a mortality disadvantage.

In our study, the determination of YPLL per 100 000 and mean YPLL provide measures of premature mortality in Asian‐American subgroups in a format that is particularly useful for studies of both population health and clinical cardiology.^14^ Population health analyses of premature mortality commonly use gross mortality rate or age‐adjusted mortality rate; however, these measures can be dominated by deaths of the elderly.^13^ YPLL more appropriately characterizes disease burden in populations because it can be weighed to take into account death at younger ages, can be adjusted to compare populations with varying age structures, and can be altered to use age limits specific to the populations under study that may differ from the majority population.^18^ These adjustments are especially useful and relevant in the study of Asian‐American subgroups.

### Limitations

Limitations of this study arise from the relatively small amount of available data about mortality and health outcomes for Asian‐Americans, especially when evaluating each subgroup separately. In the calculation for race‐specific life expectancy, we used a Census report from 2000, because it is the most recent data set published relevant to our study period, 2003 to 2012. Census data for Asian‐Americans are released only once every 4 years, or at longer intervals. The National Vital Statistics Report does not include information about Asian‐Americans, and therefore could not be used to calculate life expectancy using a life table. Census data for our study period only reported life expectancy for the non‐Hispanic Asian population. Although this should not affect our results greatly, since the proportion of Hispanic Asians is 3% in general, and just 1.5% in our study population, it includes the possibility of omitting small populations that may report as Hispanic, such as Filipino‐Americans. Other limitations include the potential for misclassification of race/ethnicity on death records that were used to gather mortality data for this study. A recent report confirmed that there was 7% to 3% misclassification for the Asian or Pacific Islander population death records.^38^

## Conclusions

Mean YPLL provides clinicians with a measure of the impact of cardiovascular disease on the life expectancy of their Asian patients, specific to each ethnic subgroup. YPLL in years per 100 000 individuals in each population during the study period gives a measure of the total burden of disease on each subgroup. Our study also provides evidence that evaluating the Asian population together as one aggregated group underestimates the burden of CVD.

## Sources of Funding

Hastings, Hu, and Palaniappan were supported by the CAUSES grant from the National Institute of Minority Health and Health Disparities Research Project (R01MD007012). Rodriguez was supported by a career development award from the National Heart, Lung, and Blood Institute (1K01HL144607).

## Disclosures

None.

## Supporting information


**Data S1.** Formula 1. Standardized age‐adjusted YPLL per 100,000 individuals
