# Supplementary material for: Years of Potential Life Lost Because of Cardiovascular Disease in Asian‐American Subgroups, 2003–2012
Source: J Am Heart Assoc. 2019 Mar 20;8(7):e010744. doi: 10.1161/JAHA.118.010744 (PMC6509739; doi:10.1161/JAHA.118.010744)
Supplement: Supplementary file 1 — Data S1. Formula 1. Standardized age‐adjusted YPLL per 100,000 individuals [file JAH3-8-e010744-s001.pdf]

SUPPLEMENTAL MATERIAL

Data S1.

Formula 1. Standardized age-adjusted YPLL per 100,000 individuals

$$YPLL = \sum(\left\{\frac{[(X_{ac})*(LE-MP_{ac})]}{P_{ac}}\right\} * S_{ac})$$

Where,

- X_{ac} = number of deaths from a specific cause (e.g. IHD), in a specific year (e.g. 2003), for a specific race/sex (e.g. Chinese American men), in a specific age category (e.g., 72-81 years, for Chinese American men, as detailed below)
- LE = Race-specific life expectancy
- MP_{ac} = mid-point of age category. Age categories for age-adjustment were created for each group. Categories were defined as 10-year increments, starting from the pre-defined life expectancy (as above) minus 10 years. The youngest age category range was larger or smaller than 10 years as the life expectancy ages for these groups were not multiples of 10. The midpoint of each age category was calculated.
- P_{ac} = total population of individuals of a certain race/sex in each age category in each year.
- S_{ac} = percentage of total individuals of a specific race/sex group in the 2000 population.
